# The Interleukin-17 Family of Cytokines in Breast Cancer

**DOI:** 10.3390/ijms19123880

**Published:** 2018-12-04

**Authors:** Joseph Antoine Salvator Fabre, Jérôme Giustiniani, Christian Garbar, Yacine Merrouche, Frank Antonicelli, Armand Bensussan

**Affiliations:** 1 Institut Jean Godinot, Unicancer, F-51726 Reims, France; fabrejoseph@yahoo.fr (J.A.S.F.); Christian.GARBAR@reims.unicancer.fr (C.G.); Yacine.MERROUCHE@reims.unicancer.fr (Y.M.); frank.antonicelli@univ-reims.fr (F.A.); 2 IRMAIC, EA7509, Université Reims-Champagne-Ardenne, 51 rue Cognacq-Jay, 51095 Reims CEDEX, France; 3 Institut Mondor de Recherche Biomédicale (INSERM U955), Equipe Immunologie et Oncogenèse des Tumeurs Lymphoïdes, Hôpital Henri Mondor, 94010 Créteil, France; jerome.giustiniani@gmail.com; 4 Institut National de la Santé et de la Recherche Médicale (INSERM) U976, Hôpital Saint Louis, 75010 Paris, France; 5 Laboratoire Immunologie Dermatologie & Oncologie, Université Paris Diderot, Sorbonne Paris Cité, UMR-S 976, F-75475 Paris, France; 6 OREGA Biotech, 69130 Ecully, France

**Keywords:** interleukin-17, breast cancer, protumor effects, antitumor effects, new target, immunotherapy

## Abstract

Breast cancer (BC) is the most common cancer in women worldwide and remains a major cause of mortality with an expected 137,000 death this year in Europe. Standard management of metastatic BC comprises hormonotherapy, chemotherapy, and targeted therapies. Cyclin dependent kinase (CDK) and mammalian target of rapamycin (mTOR) inhibitors have recently proved their efficiency in hormonal receptor expressing BC. Checkpoint proteins inhibition is being evaluated in phase 3 studies. Since inflammation is constantly present in cancers, research teams have focused their attention on the interleukin-17 (IL-17) family of proinflammatory cytokines. Preclinical experiments have reported both pro and antitumor effects depending on the conditions. In the present article, we review the accumulating evidences about the roles of IL-17 in BC and discuss whether this family of cytokines could be a new target in anticancer treatments.

## 1. Introduction

In 2018, breast cancer (BC) remains a burden for women all around the world. Considering Europe only, female BC represents 523,000 cases, standing as the first cause of cancer before the 500,000 cases of colon cancer [1]. BC management has evolved tremendously over these last 30 years. Risk factors are better understood but efficient prevention means are to be found yet. Improvement in patients screening and diagnosis has led to a drop of BC mortality. By using mammography, physicians are able to detect BC at the earliest stages, which helps allowing curability and reduce the mortality effectively [2]. This is especially true for localized BC, which can be cured in more than 80% patients [2]. Unfortunately, patient with aggressive and extended disease only survive between 3 and 12 months for most of them in the latest trials [3]. Late stages, like metastatic breast cancer, allow palliative systemic treatments only, such as chemotherapy and targeted therapies most of the time [4].

Decision-making is based on clinical assessment of the patient, tumor staging, but also on the immunohistochemical characteristics of the disease [2]. Among the 20 different histological subtypes of BC, the most frequent are the Invasive Carcinoma of No Special Type (IC-NST) and the Invasive Lobular Carcinoma (ILC) representing around 80% and 5–15% of all cases, respectively [2]. Assessing the presence of biomarkers, such as estrogen receptors (ER), progesterone receptors (PR), human epidermal growth factor 2 (HER2), and determining the proliferative index Ki67 helps defining BC subtypes, each having a different response to endocrine therapy, chemotherapy or HER2 targeted treatment such as trastuzumab [5]. Besides, groundbreaking studies of the gene expression patterns by Perou and Sorlie identified two main clusters relevant for their clinical behavior and outcome [6,7]. The largest cluster is ER-positive tumors and comprises Luminal A and B subtypes [8]. Luminal A BC are positive for ER and PR, negative for HER2, have a low Ki67 and P53 mutations rate, whereas Luminal B can either bear HER2 overexpression or not, have a lower expression of hormonal receptors but still positive, a higher rate of p53 mutations and a high expression of proliferation related genes [8]. HER2+ tumors overexpress HER2, but are negative for hormonal receptors detection [8]. Basal-like tumors present as triple negative for ER, PR and HER2 by immunohistochemistry staining, have the highest rate of p53 mutations, and express cytokeratins and proliferation genes [8]. 

Better understanding of molecular and genetic diversity of BC leads the path to personalized treatments [7]. Most recent anticancer treatments build up on biological mechanisms. Thus, favoring the molecular stratification of BC is now required to manage patient appropriately [9]. Treatments of patients with a disseminated disease rest on the combination of systemic intravenous therapies like endocrine therapy, chemotherapy, monoclonal antibodies, tyrosine kinase inhibitors (TKI) or polyadenosine phosphate ribose polymerase (PARP) inhibitors [9]. Recently cyclin-dependent kinases and mammalian target of rapamycin inhibitors have proved their efficiency in hormonal receptor expressing BC [10]. Unlike patients with an early disease, chance of cure in a metastatic setting are very poor because BC cells either inevitably develop resistance to every single agent or find shelter in organs like brain or bones, where chemotherapy does not penetrate well enough [10]. Treatments then are more likely to be evaluated in terms of progression free survival [11]. A retrospective study from SEER registries on 25,323 women diagnosed with a stage IV BC reported overall survival (OS) at 5 years and 10 years of only 26.8% and 12.8%, respectively [12]. If overall response rate remain low in immunotherapy (about 20%) [13], combination with chemotherapy may bring higher rates [14]. 

**Figure 1:**
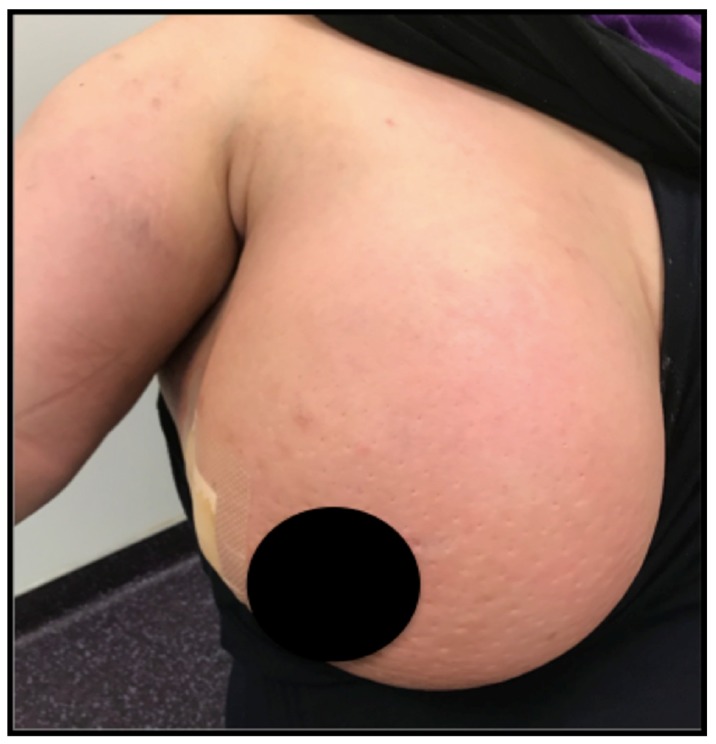
Photograph of an inflammatory breast cancer case with typical “orange skin”.

Considering cancer is frequently associated and enhanced by local inflammation, research teams have been investigating the tumor microenvironment with success [15]. Breakthrough results have been obtained with checkpoint inhibition in melanoma or nonsmall cells lung cancers (NSCLC) [16,17]. In order to improve long-term survival, recent trial in BC are also investigating checkpoint inhibitor-based immunotherapy and provide promising results especially in triple negative breast cancer (TNBC), where inflammation is preponderant, although HER2+ tumors may also be concerned (Figure 1). First results in BC presented at the 2018 American Society of Oncology meeting are promising but good, long-term outcomes only concern a minority of patients [18]. Nevertheless, those positive results remain insufficient since only a minority of patients will benefit from it, and new treatment targets are desperately needed. 

Virchow considered a relationship between inflammation and cancer early in 1863 [19]. Cancer at several stages of its development is known to be regulated by inflammation that recruits resident or circulating immune cells to modulate the tumor microenvironment [20]. These reactions are orchestrated by soluble mediators also called cytokines, secreted either by the host immune system, or by malignant cells [21]. Several interleukins have already been used as treatment or therapeutic target in oncology. IL-2 as well as IL-21, for instance have been tested in clinical trials in Melanoma and renal cell carcinoma [22]. In breast cancer, several studies have evaluated the functions of IL-1, IL-6, IL-17, IL-32 families of cytokine [23,24,25]. In the present work, we focus on the IL-17 family of cytokines. The interleukin-17 (IL-17) family of cytokines is deeply implicated in chronic inflammatory diseases and is gaining interest as an actor in cancer immunity [26]. Targeting the IL-17/IL-17R axis in breast cancer preclinical models seems to produce versatile effects depending on the manner the studies are conducted [27] (Table 1). In first place, we recall the fundamental knowledge of the IL-17/IL-17R axis, then we review the accumulating evidence about the role of IL-17 in BC, and finally we discuss whether it could be used as a therapeutic target in clinical practice. 

## 2. The IL-17/IL-17R Axis

### 2.1. The Interleukin-17 Family of Cytokine

Firstly discovered and prototypic member of the family, IL-17A also called IL-17 was initially named CTLA8 by Rouvier et al. in 1993 [28]. It was highlighted while screening a lymphoid cell gene expression library in quest for novel immune molecules. The mRNA sequence of CTLA8 had homologies with other cytokines like a 3′ untranslated region adenylate-uridylate-rich (AUR) elements and a hydrophobic N-terminal sequence [28]. Two years later, Yao et al. determined that CTLA8 could upregulate NF-kB and trigger IL-6 secretion in fibroblasts, and provoke T cell proliferation [29]. They also isolated a cDNA encoding for an unknown receptor that could bind CTLA8, and proposed to name them IL-17R and IL-17 respectively [29]. With the human genome sequencing achieved, 5 other members of the IL-17 family, IL-17B, IL-17C, IL-17D, IL-17E and IL-17F were recognized using sequence homology [30]. These cytokines play roles in immunity, in infections [31], in tumor immunity [32], in inflammatory chronic disease [33,34], in autoimmunity [35,36,37], and in atherosclerosis [38]. IL-17F is the closest to IL-17A from a sequence point of view (55% homology), is secreted by the same cell types and the genes encoding these two cytokines are both located on chromosome 6 [39]. IL-17F had its crystal structure determined first, which revealed a monomer fold typical of cysteine knot growth factors, such as Nerve growth Factors [40]. This structural motif is shared among all members of the family and is also present in bone morphogenic proteins (BMPs), transforming growth factor β (TGF-β) and platelet-derived growth factor BB (PDGF-BB) [41,42]. IL-17A and IL-17F can be secreted either as homodimers or as a heterodimer cytokine typed IL-17A/F [43,44]. Oppositely, IL-17E, also called IL-25, shares the lowest rate (17%) of similarity with IL-17A [45], compared with IL-17B (29%), IL-17D (25%), and IL-17C (23%) [46,47]. IL-17A and IL-17F are the hallmark of Th17 lymphocytes production, but not only [36], conversely to IL-17B, IL-17C, IL-17D and IL-17E, which are expressed by diverse cellular sources [48]. 

### 2.2. The Interleukin-17 Receptor

Every member of the IL-17 family signals through a specific family of receptors named interleukin-17 receptors (IL-17R) [42]. The functional receptor is a heterodimer composed by the association of 2 among the 5 different subunits (IL-17RA to IL-17RE) identified so far, each presenting as a single-pass transmembrane protein. All subunits have in common an extracellular fibronectin III domains and an intracellular “SEFIR” (similar expression to fibroblast growth factor genes and IL-17R) domain. A helix αC motif inside the SEFIR domain appears to be mandatory for interactions with Act1 [49]. Subunits combinations are specific of the IL-17 type they can match with. IL-17A, IL-17F and IL-17A/F dock to IL-17RA/RC, IL-17B and E involve IL-17RB subunit, IL-17C signals through IL-17RA/RE [50,51]. These evidences point out IL-17RA as a major partner. The receptor for IL-17D and the ligand for IL-17RD are yet to bet discovered. Signaling pathways differ between heterodimers, and they have different affinity according to the type of cytokines they bind to. IL-17F has the lowest affinity with IL-17RA and the highest affinity with IL-17RC, but IL-17A signaling produces more potent effects than IL-17F [52,53]. Two different pathways can be activated when IL-17RA/RC recognizes IL-17A. The canonical pathway activates Act1 via the SEFIR domain, which leads to E3 ligase activation and ubiquitination of TRAF6. Follows the activation of NF-kB and MAPK pathways, including ERK, p38 and JNK. This ultimately leads to the transcription of the genes of the inflammatory response. The noncanonical pathway is mediated by IkB kinase (IKKi) and the phosphorylation of Act1 at residue 311 by TBK1. The receptor complex then recruits TRAF2 and TRAF5. Then, mRNA stabilizing molecules are activated such as HuR and in parallel destabilizing factors such as ASF/SF2 are inhibited [54]. Other IL-17 receptors have been reported to recruit intracellular effectors TRAF4, TRAF6-TAK1 complex, SMURF2, DAZAP2 in different organs and settings.

### 2.3. Cellular Production of IL-17

Initially, T helpers were thought to differentiate into only two subtypes, Th1 and Th2, tough the possibility of the existence of other subtypes was considered [55]. Besides, IL-17 production could be obtained from some Th0 or Th1 clones from synovial membranes and synovial fluids of rheumatoid arthritis patients but not from Th2 lymphocytes [56]. Indeed, the scientific community, because of the discordance they observed, rapidly questioned this model [57]. In 2005, Park et al. were the first to discover a third lineage of Th cell, which could produce IL-17A [58]. Further studies indicated that these “Th17” lymphocytes could also secrete IL-17F as well as IL-21, IL-22 and GM-CSF [59,60,61]. Commitment to the Th17 pathway is a two-step process. First naïve T cells (Th0) have to be activated by dendritic cells concomitantly to, IL-6 and TGF-β (or IL-1β, IL-6 and IL-23) exposure. Thus IL-23R gene becomes upregulated [62]. Then, IL-23 needs to be present to stimulate IL-23R, and to launch intracellular signal through STAT3 and TYK3 in order to reveal full aptness [63]. Lymphocyte Th17 polarization following cytokine immersion takes place in secondary lymphoid organs, during antigens presentation [64]. Similarly to Th1 and Th2 lymphocytes, activated Th17 recruit a signature transcription factor named RAR-related orphan receptor gamma (RORγ) responsible for the transcription of several specific DNA loci critical for Th17 differentiation [65]. 

Recent reports strongly support secretion of IL-17A and IL-17F by other cellular sources than Th17. Huber et al. observed CD8+ T cytotoxic cells could be an important source of IL-17A [66]. Innate tissue resident cells (ITRC) have also been reported to participate to IL-17 production [38]. This last subset comprises γδT cells, type 3 innate lymphoid cells (ILC3), NKT cells and “natural” Th17 [67,68]. These cells are posted at barrier sites and can respond quickly to germs and tissue aggression with IL-17 synthesis [69]. Biologically, they harbor the chemokine receptor CCR6 and rely upon IL-23 exposure and RORγ activation to produce IL-17. IL-17 production was also associated with infiltrated innate immune cells. Actually, IL-17 production has been mainly attributed to neutrophils in bullous pemphigoid, a skin autoimmune bullous disease [36]. Possibility of IL-17A expression by macrophages, and mast cells remains under discussion. Phagocytosis mechanism probably explains why IL-17 in these myeloid cells is measured at levels significantly below than those detected in Th17 cells. Some authors even observed mast cells could engulf extracellular IL-17A from the environment via receptor-mediated endocytosis, store it, and release it during tissue inflammation [70]. In addition, IL-17A can be liberated as extracellular traps by either neutrophils or mast cells [71].

IL-17E has at least 80% of divergence with IL-17A, B and C and can be produced by hematopoietic and nonhematopoietic cells [72]. The expression of IL-17E by mast cells and alveolar macrophages has been observed in a mice model of asthma [73]. In humans, detection of IL-17E mRNA was detected in eosinophils and basophils of allergic subjects [74]. Still in the context of infection or allergy, expression of IL-17E is also possible by tissue stromal cells like lung epithelial cells and murine primary type II alveolar epithelial cells [75]. Thus, in asthmatics, concentrations of IL-17E were significantly enhanced in the bronchial mucosa and dermis when challenged by antigen. Noteworthy, in this study, endothelial cells were also positive for IL-17E immunoreactivity [76]. These results show that IL-17E-induced inflammation is pleiotropic and characterized mostly by an eosinophil infiltrate, whereas IL-17A and IL-17F production rather involve neutrophils. Besides, this study confirms deep implication in the deregulation of Th2 responses seen in chronic allergy diseases [77]. 

IL-17C is less known than IL-17A, IL-17F and IL-17E, and its roles in inflammation or infection are only beginning to be understood. It has the particularity to be secreted by epithelial cells I in response to toll like receptor 2 (TLR2) and TLR5 ligand binding and stimulation by IL-1β and TNF-α [78]. Additional cell types comprising CD4+ T lymphocytes, dendritic cells and macrophages have been reported to be sources of IL-17C, though at lesser levels [79,80]. 

IL-17B seems pleiotropic, and has been detected in various cell types such as neurons, intestinal epithelial cells chondrocytes, and BC cells [80]. Finally, IL-17D can be expressed in various organs comprising adipose tissue, brain, heart, lung pancreas and skeletal muscle, and seems to be limited to naïve CD4+ T cells and B cells [81]. 

## 3. Roles of IL-17 in Breast Cancer Models

It is now well known that tumor cell detour the inflammatory process to progress, metastasize and evade destruction by immune cells [82]. Inhibitors of IL-17/IL-17R have been quite successful in inflammatory diseases, especially in psoriasis where tremendous responses in advanced disease and a real benefit for patients were obtained [83]. Breast cancer presents clinically under an inflammatory form (Figure 1) in 5% of cases and some subtypes such as TNBC or HER2+ are known to heavily rely on inflammation [84]. Therefore, researchers first tried to detect IL-17 family members’ expression in the tumor environment. 

In a both preclinical and clinical study, Zhu et al. examined tissue material from 4 Scarf Bloom and Richardson (SBR) grade II and 15 SBR III tumors (molecular subtype was not mentioned) [85]. Peritumoral area was strongly stained for IL-17 in 8 of the grade III lesions but none of the grade II [85]. Regarding the identity of the IL-17+ cells, they corresponded to CD68+ macrophages [85]. Notably, no staining was observed on the BC cells [85]. When assessing the impact of IL-17 on BC cell lines (Luminal A MCF-7 and T47D, triple negative MDA-MB435 and MDA-MB231), they observed that metalloproteinase (MMP)-dependent invasion of matrigel was increased in MDA-435 and MDA-MB231, but not on the other cell lines when supplemented with IL-17 [85]. Several other studies support a protumor role of IL-17 in BC and are listed in Table 1 [45,86,87,88,89,90,91,92]. In a murine model grafted with TNBC, Du et al. observed that intratumoral level of IL-17 was increased and correlated with the expansion of the disease [87]. They also assessed the impact of IL-17 on the TNBC grafts and reported a stimulatory effect on growth and on angiogenesis with microvascular density [87]. Another report indicated that IL-17 promoted tumor graft development and directly inhibited apoptosis in 4T1, MDA-MB-231 and EM6 BC cell lines in a TGF-β dependent manner [90]. Further, it is shown that this effect was reversible under IL-17R suppression using knock out technique [90]. Stimulation of MCF7 BC cells by IL-17A induced MEK, ERK, JNK, cJun and STAT3 phosphorylation and lead to increased cell proliferation as assessed with BrdU incorporation [35]. Concordantly, tyrosine kinase inhibitors drastically reduced colony formation, and even abolished it completely in siRNA-IL-17A cells. The author hypothesized that MAPK activation was initiated by recruiting tumor progression locus 2 (TPL2), but they could only observe it on epidermal cells [35]. Our team has been investigating IL-17 role in breast cancer for several years [86,89,93,94]). We demonstrated that tumor infiltrated lymphocytes (TIL) isolated from BC biopsies secreted pathophysiological IL-17A. Consistent with Nam et al., we demonstrated that recombinant IL-17A activated the ERK1/2 pathway and thus promoted resistance to docetaxel-based chemotherapy in various cell lines [86]. Proliferation of ER+ T47D cells was significantly enhanced as well as migration and invasion of MCF7. Besides, physiological IL-17A had the same proliferative and protective effects as recombinants, which were partially abrogated by anti-IL-17A OREG-203 antibody [86]. We then dug deeper in the signaling pathway and reported that the recruitment of c-RAF and S6 kinases by both IL-17A and IL-17E, confers chemoresistance and to ultimately generates low molecular weight cyclin E [93], which is clinically associated with poor prognosis [95]. A well-known mechanism of chemoresistance and/or radioresistance is the recruitment of members of the epidermal growth factor (EGF) family (HER) of receptors [96]. We reported in 2016 that IL-17 signaling probably had crosslinks with HER1 [89]. Indeed, stimulation of TNBC cell lines with IL-17E induced the phosphorylation of HER1 and triggered pHER1 and pSTAT3 translocation to the nucleus, and promoted resistance to TKI [89]. Noteworthy, this mechanism was abolished by anti-IL-17RB antibodies [89]. IL-17B has also shown protumor roles in BC. Huang et al. observed that activation of IL-17B/IL-17RB signaling had protumor effects in vitro and in vivo via TRAF6 and NF-kB [97]. Conversely, in our last study, IL-17B produced IL-17RB-mediated resistance to paclitaxel in cell lines BT20, MDA-MB-468 and MCF7 [94]. Similar findings were obtained in vivo, in an experimental model of xenografted nude mice [94]. Subcutaneously injected IL-17RB or MAPK antibodies inhibited cell resistance to chemotherapy [94]. Meanwhile, IL-17B did not affect cell or tumor proliferation [94]. Apart from the direct effects on BC cells, an indirect role of IL-17 on neutrophils has been reported by Coffelt et al. [98]. In mice, the authors observed that γδ T cells were the main source of IL-17A, which release induced neutrophils to suppress CD8+ T lymphocytes, and subsequently metastases development [99]. Depletion of neutrophils or γδ T cells did not affect the primary tumor progression, but significantly reduced pulmonary and lymph node metastasis [99]. Besides neutrophils, tumor-infiltrated Th2 cells and macrophages may be affected by IL-17 cytokines as described in [99]. IL-17E was expressed by tumor infiltrating CD4+ T cells and macrophages, and inhibition of IL-17E caused reduced tumor infiltration by both types of leucocytes, which was associated with a significantly disabled lung metastasis formation [99]. Some authors proposed that the IL-17A positive impact on metastasis rate was related to its effects on MMP-11 upregulation in mononuclear inflammatory cells [88,100]. Concordantly, Roy et al. reported in a pro arthritic mouse model that IL-17A induced metastases to the bones and lungs via upregulation of stromal cell derived factor 1 (SDF-1) directly or via IL-6 and M-CSF secretion promotion [91,92]. 

In opposition, research teams have shown in certain conditions anti-tumor effects of IL-17 [101,102,103]. In cancer xenograft mouse models (CD1 nude and SCID), a variety of tumors (melanoma, nonsmall cell lung cancer, colon adenocarcinoma, pancreatic adenocarcinoma and breast adenocarcinoma) were analyzed with respect to IL-17 expression [101]. Human IL-17E (hIL-17E) or mouse IL-17E (mIL-17E) was administered using different routes [101]. Every cancer type including BC cell lines MDA-MB-435 was reported to have significantly reduced size upon IL-17E stimulation as compared with control. Besides, antitumor activity of IL-17E was potentiated when combined with cisplatin or taxol [101]. This effect was nullified in T and B cells deficient mice but not in those lacking only T lymphocytes suggesting a critical role of B lymphocyte in the antitumor effect of IL-17E [101]. Furuta et al. identified IL-17E as responsible for the cytotoxicity they observed when adding the conditioned medium (CM) from nonmalignant mammary epithelial cell (MEC) to breast cancer cell cultures [102]. Indeed, when they cultured BC cell lines with purified IL-17E from MEC, or injected it in murine model of BC grafts, they obtained respectively a drop in the number and size of the colonies, and a strong antitumor effect [102]. Recent study by Ma et al. added that IL-17A could directly induce myeloid-derived suppressor cell lines differentiation, apoptosis and reduced proliferation indicating that enhancing IL-17 pathway may revive immune response [103]. 

## 4. Discussion

**Figure 2:**
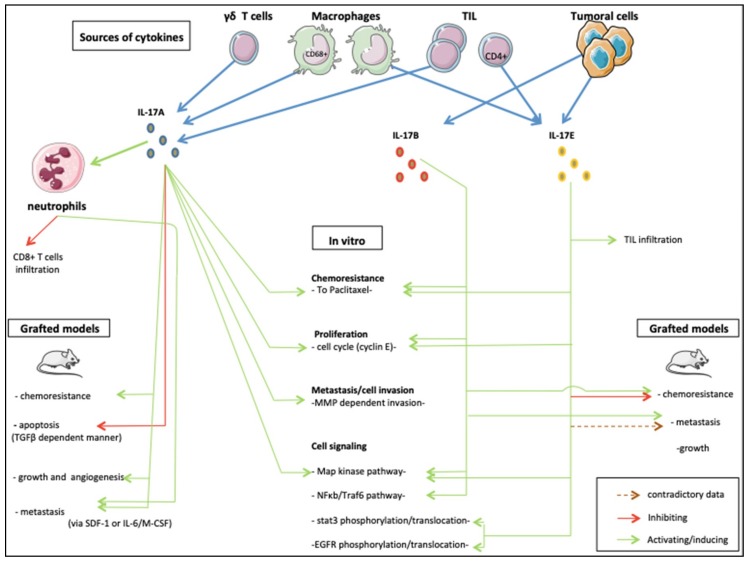
The roles of IL-17 family members in breast cancer.

From its discovery to now, the IL-17 family of cytokines has revealed critical roles in inflammatory disease and seems deeply implicated in cancer development [105]. Here, we synthetized the results of studies published to date about the roles of the IL-17 family cytokines in BC cells. Of the 6 members, only IL-17A, IL-17B and IL-17E were reported to have oncogenic effects (Figure 2). Protumoral effects on proliferation, angiogenesis, invasion, migration and resistance to treatments have been observed in a variety of cell lines and mouse models. These effects may be direct via widely detected IL-17Rs signaling through MAPK and NF-kB recruitment. IL-17 cytokines also act indirectly on immune and nonimmune peritumoral cells’ cytokine secretion. In certain conditions, an antitumor role was also described. The same duality has been observed in other tumor types making IL-17 a “double-edged sword” in oncology [106]. Honorati et al. for instance, described improved susceptibility of U-2 osteosarcoma to NK cell lysing ex vivo [107]. This observation concurs with the anti-tumor effects on sarcoma of IL-17D in vivo via NK cells recruitment [108]. In opposition, IL-17A has been reported to promote tumor growth either indirectly via the production of other pro inflammatory cytokines by immune cells, or directly via stimulation of tumor stem cells in glioma models [109]. Protumor effects of IL-17 seem also preeminent in cervical and lung cancer using different action modes [110]. From a cellular point of view, IL-17A promoted invasion and migration, [111] potentially via MMP expression through p38/NF-kB pathway activation [112]. In vivo, experiments are converging towards tumor growth stimulating roles [113] possibly through angiogenesis stimulation by VEGF [114]. Nevertheless, cancer types investigated in these studies have far different behavior than BC. Prostate cancer (PrC) on the other hand, share numerous similarities with BC, in particular a vast diversity of phenotypes, biology and both cancer types build on steroidal stimulation to develop and expand [115]. Zhang et al. observed that IL-17A could promote prostate adenocarcinoma formation and development in mice models [116], and later suggested the MMP-7-induced epithelial-to-mesenchymal transition as a possible mechanism induced by IL-17A [117]. The same team also reported in a model that IL-17A in combination with insulin/IGF1 enhanced VCAM-1 increased the expression of endothelial cells and thus increased PrC cells adhesion [118]. 

Overall, conflicting outcomes may simply result of different experimental conditions and variation of the environment. Timing of BC cells exposure to IL-17, type of cells present, and stage of the disease may also be of importance [119]. Nevertheless, data converge to present IL-17/IL-17R signaling as a potential target especially in inflammatory forms of BC, embodied by triple negative and Her2+ subtypes. Besides, from the now consequent experiences in psoriasis, IL-17 pathway disrupting antibodies such as secukinumab, ixekizumab, ustekinumab and brodalumab have proven to be safe to use [120]. Therefore, we look forward to developing strategies integrating the cytokines of the IL-17 family in anticancer therapies. 

## 5. Patents

The authors own possession of the photograph in Figure 1 and have informed consent of the patient. 

Combinations therapies for treatment of cancer. WO2017194554.

## Figures and Tables

**Table 1:** Studies reporting the roles of IL-17 cytokines in breast cancer in vitro and/or in vivo.

Type of IL-17	Type of Study	Breast Cancer Cell Lines	Effect of IL-17 Exposure	Murine Model	Mechanism	Reference
IL-17A	Clinical and preclinical	No	Anti	BALB/c Nude	Induction of differentiation and apoptosis, inhibition of proliferation of MDSC via STAT3	[103]
IL-17A	Preclinical	No	Pro	KEP, Tcrδ−/−	Production of IL-17A by γδ T cells induces neutrophils to suppress CD8+ T Cells and promotes distant metastases.	[98]
IL-17A	Clinical and preclinical	MCF7, T47D, BT20, MDA-MB468, MD-MB157, MDA-MB231	Pro	No	Activation or ERK1/2 pathway induces proliferation, migration, invasion and chemoresistance	[86]
IL-17A	Clinical	No	Pro	No	IL-17A associated to MMP-11+ mononuclear infiltrating cells which are correlated to metastasis	[88,100]
IL-17A	Preclinical	MCF7	Pro	No	Activation of MAPK: MEKK, ERK, JNK, cJun, STAT3.Cell proliferation.	[35]
IL-17A	Preclinical	MA782, 4T1	Pro	BALB/c	Increase in tumor volume and microvascular density	[87]
IL-17A	Preclinical	4T1, PyV MT cell line	Pro	PyV MT, arthritic PyV MT	Upregulation of SDF1, IL-6, G-CSF. Promotion of bone and lung metastases	[91,92]
IL-17A	Clinical and preclinical	MCF-7, T47D, MDA-MB435, MDA-MB231	Pro	No	Recruitment of macrophages, activation of MMP	[85]
IL-17A	Preclinical	4T1, MDA-MB231, EM6, MDA-MB435, Hs578t	Pro	BALB/c	TGF- β dependent tumor growth, inhibition of apoptosis	[104]
IL-17E	Clinical and preclinical	No	Pro	MMTV-PyMT	Production of IL-17E by tumor- infiltrating macrophages	[99]
IL-17E	Preclinical	MCF7, MDA-MB468, MDA-MB 435-S, MDA-MB231,SKBR3, T47D, ZR75, Hs578t, HCC1937, MDA-MB175-7	Anti	Nude	Induction of apoptosis, decrease in colony formation and tumor growth	[102]
IL-17E	Preclinical	MDA-MB-435	Anti	CD1-nude	Decrease in tumor volume. B cells mandatory	[101]
IL-17A and Il-17E	Preclinical	MDA-MB468, BT20, IJG-1731	Pro	No	Activation of STAT3, PYK-2, Src and HER-1. Nuclear translocation of pSTAT3 and pHER-1. Resistance to TKI.	[89]
IL-17A and IL-17E	Clinical and preclinical	T47D, MCF7, BT20, IJG-1731	Pro	No	Activation of cRAF and S6 kinases. Chemoresistance and generation of LMWCE	[93]
IL-17B	Clinical and preclinical	BT20, MDA-MB-468, MCF7	Pro	Nude	Resistance to paclitaxel in cell lines and xenografts via ERK pathway. Upregulation of BCL2.	[94]
IL-17B	Clinical and preclinical	MCF7, MDA-MB-157, MDA-MB-231, MDA-MB-361, MDA-MB-468, SKBR3, SKBR3-hr	Pro	NOD/SCID/γ^null^	Promotion of proliferation and tumor growth through IL-17RB via NF-kB and TRAF6	[97]

## Abbreviations

BC	Breast cancer
CDK	Cyclin dependent kinase
mTOR	Mammalian target of rapamycin
IL	Interleukin
NSCLC	Nonsquamous cells lung cancer
IC-NST	Invasive carcinoma of non special type
ILC	Invasive lobular carcinoma
ER	Estrogen receptor
PR	Progesterone receptor
HER	Human epidermal growth factor receptor
TKI	Tyrosine kinase inhibitors
PARP	Poly adenosine diphosphate ribose polymerase
SEER	Surveillance epidemiology and end results
TNBC	Triple negative breast cancer
OS	Overall survival
CTLA8	Cytotoxic T lymphocytes associated lymphocytes 8
AUR	Adenylate uridylate rich
NF-kB	Nuclear factor kappa B
cDNA	Complentary desoxyribonucleic acid
TGF-β	Transforming growth factor β
IL-17R	Interleukin-17 receptor
SEFIR	Similar expression to fibroblast growth factor genes and IL-17R
TRAF	Tumor necrosing factor receptor associated factor
MAPK	Mitogen-activated protein kinase
ERK	Extracellular signal-regulated kinase
p38	p38 kinase
JNK	c-Jun N-terminal kinase
IKKi	I kappa B kinase
TBK1	TANK binding kinase 1
mRNA	Messenger ribonucleic acid
HuR	Human antigen R
ASF/SF2	Serine/arginine rich splicing factor 2
TAK1	Transforming growth factor β activated kinase 1
SMURF2	Smad ubiquitination regulatory factor
DAZAP2	Deleted in azoospermia associated 2
GM-CSF	Granulocyte-macrophage colony-stimulating factor
STAT	Signal transducer and activator of transcription
TYK	Tyrosine kinase
RORγ	RAR-related orphan receptor gamma
ITRC	Innate tissue resident cells
ILC3	type 3 innate lymphoid cells
CCR	Chemokine receptor
SBR	Scarf Bloom and Richardson
BrdU	Bromodeoxyuridine
siRNA	Small interfering ribonucleic acid
c-RAF	RAF proto-oncogene serine/threonine-protein kinase
STAT	Signal transducer and activator of transcription
TPL2	Tumor progression locus 2
TIL	Tumor-infiltrating lymphocytes
MMP	Matrix metalloproteinase
SDF-1	Stromal derived factor 1
M-CSF	Macrophage colony-stimulating factor
SCID	Severe combined immunodeficiency
CM	Conditioned medium
MEC	Mammary epithelial cell
MDSC	Myeloid-derived suppressor cells
LMWCE	Low molecular weight cyclin E
MEKK	MAPK/extracellular signal-regulated kinase kinase
PrC	Prostate cancer
